# Assessing the suitability of long non-coding RNAs as therapeutic targets and biomarkers in SARS-CoV-2 infection

**DOI:** 10.3389/fmolb.2022.975322

**Published:** 2022-08-16

**Authors:** Yichen Zhong, Caroline L. Ashley, Megan Steain, Sandro Fernandes Ataide

**Affiliations:** ^1^ School of Life and Environmental Sciences, University of Sydney, Sydney, NSW, Australia; ^2^ School of Medical Sciences, Faculty of Medicine and Health, The University of Sydney, Sydney, NSW, Australia

**Keywords:** COVID-19, SARS-CoV-2, cytokine release syndrome, lncRNA profiling, NLRP3 inflammasome, IL-6, IFN-I, IL-17

## Abstract

Long non-coding RNAs (lncRNAs) are RNA transcripts that are over 200 nucleotides and rarely encode proteins or peptides. They regulate gene expression and protein activities and are heavily involved in many cellular processes such as cytokine secretion in respond to viral infection. In severe COVID-19 cases, hyperactivation of the immune system may cause an abnormally sharp increase in pro-inflammatory cytokines, known as cytokine release syndrome (CRS), which leads to severe tissue damage or even organ failure, raising COVID-19 mortality rate. In this review, we assessed the correlation between lncRNAs expression and cytokine release syndrome by comparing lncRNA profiles between COVID-19 patients and health controls, as well as between severe and non-severe cases. We also discussed the role of lncRNAs in CRS contributors and showed that the lncRNA profiles display consistency with patients’ clinic symptoms, thus suggesting the potential of lncRNAs as drug targets or biomarkers in COVID-19 treatment.

## Introduction

The human genome produces ∼20,000 protein coding transcripts corresponding to a small portion of the whole cell transcriptome (Piovesan et al., 2019). The majority of RNA transcripts do not encode an open reading frame (ORF) and are referred as non-coding RNAs (ncRNAs) which include the well-known transfer RNAs (tRNA), microRNAs (miRNA) and long non-coding RNAs (lncRNAs) that are over 200 nucleotides in length. LncRNA can be transcribed from the non-coding regions between genes or regions close to/within the coding regions, such as introns and enhancers in either or both sense and antisense manners (Ma et al., 2013). LncRNA has more in common with messenger RNA (mRNA) than other shorter ncRNA. mRNA and lncRNA are both transcribed by RNA polymerase II (RNA pol II), and lncRNAs also undergo 5′-capping, polyA tailing and co-transcriptional splicing, but at lower efficiency than mRNA (Ransohoff et al., 2018).

**FIGURE 1:**
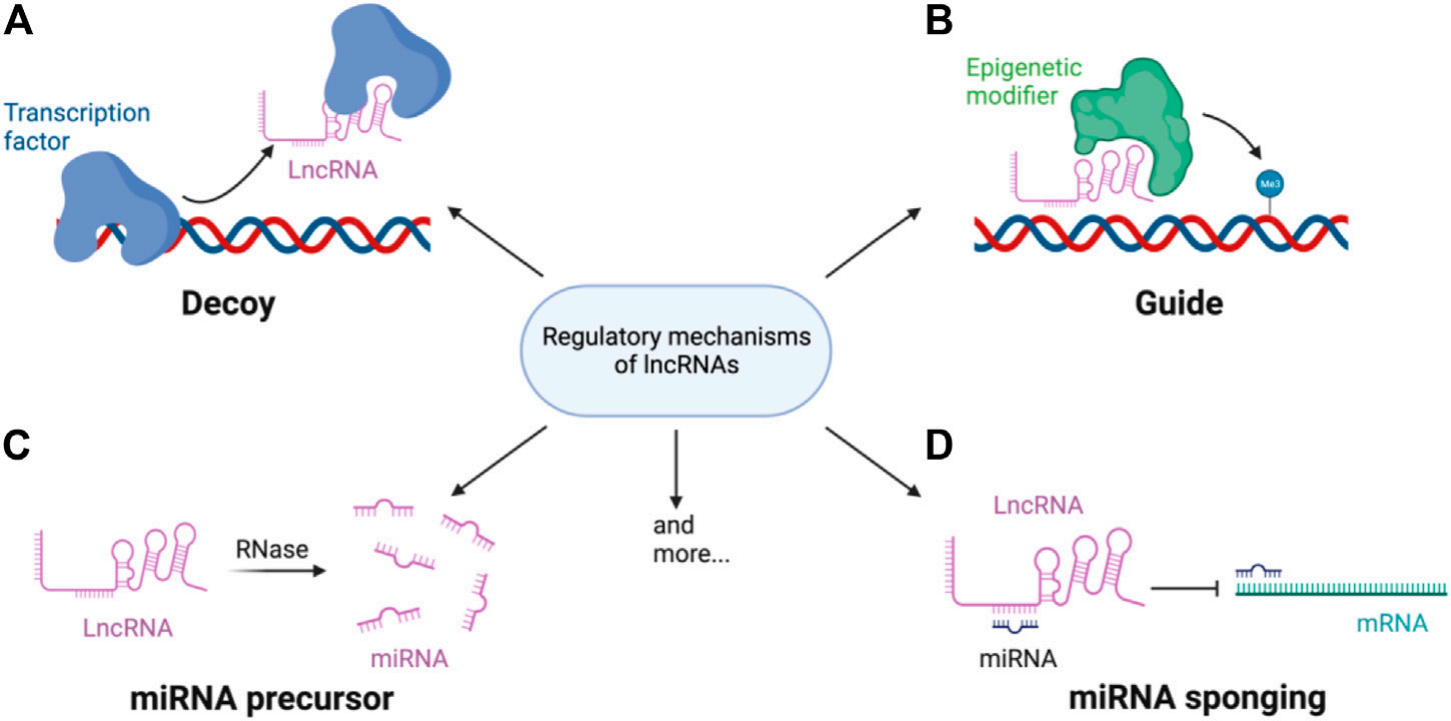
Regulatory mechanisms of lncRNA. LncRNAs regulate gene expression through **(A)** decoying or guiding transcription factors and/or **(B)** epigenetic modifier to the target loci. LncRNAs can also be **(C)** processed into mature miRNAs or **(D)** bind miRNA to sponge the miRNAs miRNA mediated gene repression or mRNA degradation.

Functionally, lncRNAs are mostly regulatory and can work in *cis* (regulate genes in close genomic proximity to their loci) or in *trans* (act in cytosol or on genomic regions distal from their transcription sites) (Figure 1). *cis*-lncRNAs are more of a transcriptional regulator than *trans*-lncRNA and function through transcriptional interference or chromatin modification. Due to their sequence complementarity and/or similarity, they can be tethered to loci of the target genes and influence RNA pol II occupancy by signaling/decoying transcription factors (TFs), enhancing chromatin looping or providing scaffolds for ribonucleoprotein complexes (Bhat et al., 2016). *cis*-lncRNAs can also recruit epigenetic modifiers to the loci and alter the active state of the genomic region. For example, polycomb repressive complex 2 (PRC2) is recruited by the lncRNA X-inactive specific transcript (XIST) to silence one of the X chromosomes during development (Maenner et al., 2010). *trans*-lncRNA, in addition to these mechanisms, can also translocate out of the nucleus and regulate miRNA and even protein activities. Some lncRNAs are miRNA precursors which can be digested to directly increase miRNA levels, whereas some regulate miRNA transcription (Sun et al., 2020). However, the most well-studied mechanism of gene regulation by lncRNAs is its ability to act as a ‘sponge’ by binding to miRNA and preventing their mRNA targeting (Lopez-Urrutia et al., 2019). LncRNA is present in almost every cellular process, including cell cycle progression and cell differentiation, as well as in responses to intra- and extracellular stimuli.

In December 2019, an outbreak of pneumonia caused by a novel severe acute respiratory syndrome coronavirus 2 (SARS-CoV-2) erupted in Hubei Province of China. The disease, given the name coronavirus disease 2019 (COVID-19), quickly started spreading and eventually became a global pandemic. Most COVID-19 patients are able to recover, but some severe cases may develop life-threatening acute respiratory distress syndrome (ARDS) and multi-organ dysfunction. Studies have shown that ARDS is highly related to a sharp increase in the level of pro-inflammatory cytokines, termed cytokine release syndrome (CRS) (Pasrija and Naime, 2021). Cytokines are small molecules responsible for signaling and controlling cell growth, differentiation, migration and antibody production in the immune system. Common cytokines include interferons (IFNs), interleukins (ILs), transforming growth factors (TGFs) and tumor necrosis factors (TNFs), and they can be pro-inflammatory or anti-inflammatory. Pro-inflammatory cytokines, such as IL-1β, IL-6, and TNF-α, are predominantly produced by activated macrophages and increases inflammatory reactions, whereas anti-inflammatory ones including IL-4, IL-10 and TGF-β suppress pro-inflammatory responses and prevent auto-immune damage (Zhang and An, 2007). Cytokine release is a crucial step in the innate immune defense mechanism, however the response induced by SARS-CoV-2 in CRS is dysregulated systemic hyperinflammation which eventually leads to lung injury and respiratory failure, sometimes before eliminating the virus.

Throughout 2019‒2022, SARS-CoV-2 has been undergoing constant mutation, with the Omicron variant and its subvariants being the current (at the time of writing) dominant strains of the globe. Early assessment in South Africa reported lower hospitalization rates and fewer severe cases, comparing to the Delta infection period (Wolter et al., 2022); some preprints have also claimed less severe symptoms in Omicron-infected rodent models (Bentley et al., 2021; Halfmann, 2022). On the other hand, Imperial College London has released data showing no evidence of diminished hospitalizations in Omicron vs Delta infection comparisons (Ferguson et al., 2021). Recently, a large study (preprint) covering more than 130,000 COVID-19 patients in the USA suggested that Omicron is just as deadly as all previous variants and the lower apparent severity was likely due to higher vaccination rates and a younger infected population on average (Strasser et al., 2022). However, considering the fast mutation rate and increasing immune evasion of Omicron subvariants [the latest Omicron BA.4 and BA.5 even escape antibodies elicited by BA.1 infection (Xie et al., 2022)], continuous studies on COVID-19 severity and treatments are necessary for global healthcare systems and preventing death in high risk groups (Mohsin and Mahmud, 2022).

LncRNA is heavily involved in the activation and/or differentiation of cytokine secreting cells (Carpenter and Fitzgerald, 2018), which undergo dramatic changes in their proteome and transcriptome in response to SARS-CoV-2 infection (Bojkova et al., 2020; Singh et al., 2021). In this review, we discussed the relationship between lncRNA expression profile and the severity of COVID-19 patients, and the role of lncRNAs in three main contributors of CRS. We found that lncRNA detected in patients’ blood cells and other tissue samples are relatively consistent with their clinical performance, making lncRNA expression patterns a potential biomarker indicating the inflammatory state of the patient. Thus, the understanding of lncRNAs and their roles in immune responses, especially cytokine secretion, could hopefully open a new gate for developing new therapies or drug targets for cytokine release syndrome (CRS) and decreasing mortality caused by SARS-CoV-2 infection.

## LncRNA expression during SARS-CoV-2 infection

### General and SARS-CoV-2-specific changes in lncRNA expression profile

As the primary target of SARS-CoV-2, lung tissues have been the primary focus for studies into changes in lncRNA expression during COVD-19 infection. Using RNA-seq, Vishnubalaji et al. identified 155 upregulated and 195 downregulated lncRNAs in normal human bronchial epithelial cells (NBHE) during COVID-19 infection, whereas Moazzam-Jazi et al. found 207 differentially expressed lncRNA in bronchoalveolar lavage fluid (BALF), of which 50% are upregulated (Vishnubalaji et al., 2020; Moazzam-Jazi et al., 2021). Two specific lncRNAs, metastasis associated lung adenocarcinoma transcript 1 (MALAT1) and nuclear paraspeckle assembly transcript 1 (NEAT1), show significant increase in expression in both NBHE and BALF. These two lncRNAs are known for their activation of innate immune responses and triggering pro-inflammatory cytokine secretion from M1 macrophages (Cui et al., 2019; Zhang P. et al., 2019c; Li Y. et al., 2020b and Wang and Guo, 2020), thus high abundance of MALAT1 and NEAT1 in lung samples could be associated with the inflammation in the local region.

Outside the lungs, lncRNA expression profiles in COVID-19 patients’ peripheral blood samples also display differences compared to healthy controls. In an RNA-seq study of total RNA extracted from peripheral blood, gene ontology (GO) functional enrichment indicates that the most differentially expressed lncRNAs are involved in cell signaling pathways and protein/RNA metabolism, influencing processes such as ion fluxes, protein phosphorylation and protein/RNA degradation (Wu et al., 2021). Consistently, signaling pathways and metabolism are also highlighted after Kyoto encyclopedia of genes and genomes (KEGG) enrichment. The altered lncRNA expression pattern was seen to affect nuclear factor kappa B (NF-κB) and T cell activation signaling pathways the most, as well as anabolism of substances such as the biosynthesis of steroid, ubiquinone and terpenoid-quinone (Wu et al., 2021). However, total RNA extraction would mix lncRNA from both peripheral blood mononuclear cells (PBMCs) and plasma, which could display different RNA landscapes.

The majority of altered lncRNAs from patients’ PBMCs are related to immune processes or the cell cycle, and influence protein coding genes in *cis*- or *trans*-acting manners. The lncRNA CCAAT enhancer-binding protein alpha divergent transcript (CEBPA-DT) is one such *cis*-lncRNA (Moazzam-Jazi et al., 2021). It enhances the expression of transcription factor CEBPA, which suppresses interferon gamma (IFN-γ) expression in T cells (Tanaka S. et al., 2014a). GO enrichment also identified clusters of *trans*-lncRNAs that are relevant to mitosis, chromosome segregation and other cyclin A/B1/B2-associated events in patients’ PBMCs, which could be related to viral infection induced cell cycle arrest and proliferation of immune cells (Moazzam-Jazi et al., 2021). On the other hand, cell-free plasma RNA profiling showed that only a small portion of total plasma RNAs (1.3%) are lncRNAs, and they regulate cytokine secretion by sponging miRNAs present in the plasma. For example, upregulation of the lncRNA GJA9-MYCBP was detected in COVID-19 patients. This lncRNA contains let-7 miRNA binding sites and thus could potentially sponge let-7 miRNAs and indirectly increase the expression levels of IL-6 and IL-6R, contributing to CRS (Wang et al., 2022).

The differential expression of lncRNA may be due to the hijacking mechanisms of SARS-CoV-2, rather than the host’s antiviral responses. Although the viral-host protein-protein interaction network and the associated proteome changes have been the center of many studies (Gordon et al., 2020; Stukalov et al., 2021), evidence of direct association between viral RNA/proteins and host lncRNA is still limited, possibly as an effect of the low abundance of lncRNAs in cells. However, these proteome data can still provide insights to lncRNA differential expressions indirectly caused by viral hijacking. For example, TGF-β receptor 2 (TGFBR2) was found in association with SARS-CoV-2 ORF8 (Stukalov et al., 2021), and its inactivation in tumor-initiating hepatocytes led to an increase in a lncRNA called H19 (Zhang J. et al., 2019a). Interestingly, H19 was also found dramatically upregulated in BALF of COVID-19 patients (Moazzam-Jazi et al., 2021). Since H19 is well-known for its role in inflammatory responses [reviewed in (Shi et al., 2020)], the sharp increase in H19 is more likely related to its function of stimulating IL-1β, IL-6 and IL-17 production (Hu et al., 2019; Zhang et al., 2020), and ORF8 hijacking might just be one of the many contributors to the outcome. On the other hand, a contradictory example is the lncRNA small nucleolar RNA host gene 6 (SNHG6). SNHG6 interacts with up-frameshift protein 1 (UPF1) to mediate the TGF-β/Smad pathway in hepatoma cells (Chang et al., 2016). UPF1 is one of the interactors of SARS-CoV-2 Nucleocapsid (N) protein (Gordon et al., 2020) and since SNHG6 is only slightly varied in patients’ BALF (Moazzam-Jazi et al., 2021), the cause of this change in expression is ambiguous. Therefore, the influence of viral hijacking mechanisms on lncRNA expression changes needs to be cautiously considered when determining if it is a suitable biomarker candidate.

The effect of SARS-CoV-2 on lncRNA could be different to other types of coronavirus infection. The lncRNA eosinophil granule ontogeny transcript (EGOT), a negative regulator of the type-I interferon (IFN-I) response (Carnero et al., 2016), shows a much greater upregulation in SARS-CoV-2 infection compared to Middle East Respiratory Syndrome coronavirus (MERS-CoV) infection, as seen in the Calu3 lung cancer cell model (Mukherjee et al., 2021). Similarly, wound and keratinocyte migration–associated lncRNA 2 (WAKMAR2), which restricts inflammatory chemokine production in keratinocytes (Herter et al., 2019), is upregulated in SARS-CoV-2 but not in MERS-CoV infected cells (Mukherjee et al., 2021). Since WAKMAR2 expression can be induced by TGF-β (Herter et al., 2019), a higher level of this lncRNA could be an indication of aberrant cytokine environment.

Overall, lncRNAs undergo differential expression in response to SARS-CoV-2 both generally and virus-specifically. They mainly function by regulating the protein-coding mRNA or sponging regulatory miRNA, thus altering the expression of proteins involved in immune cell differentiation and cytokine production. In addition, these lncRNAs also induce pro-inflammatory responses by activating processes that are related to CRS. However, comparing COVID-19 patients and health controls without considering severity cannot fully reveal the role of lncRNAs in CRS, which is more commonly observed in severe cases.

### LncRNA profile in severe vs. non-severe cases

The severity of COVID-19 has been associated with several factors, including age, gender, selected comorbidities and genetic variants (Fang et al., 2020; Noor and Islam, 2020; Choudhary et al., 2021; Fadl et al., 2021). On the molecular level, severe cases show differences in their immune response to infection compared to mild and moderate cases.

Severely affected patients also display a different lncRNA profile in their PBMC compartment. The results of Moazzam-Jazi et al. showed that 83% of lncRNAs in PBMCs of COVID-19 patients are downregulated, but Cheng’s study claimed that the underlying expression changes are more complicated than initially believed (Cheng et al., 2021). By splitting the patients into severe and non-severe groups and comparing their lncRNA profiles with that of healthy controls, Cheng et al. found that the non-severe cases have more upregulated lncRNAs than controls, whereas lncRNAs in severe patients display an overall downregulated trend compared to control and non-severe cases (Cheng et al., 2021). For example, XIST and LINC01619 are upregulated in non-severe cases yet the expression levels are not as high in severe cases. Likewise, LINC00278 is weakly downregulated for non-severe cases but significantly upregulated in correlation with the severity of infection. LINC00278 was recently reported to promote apoptosis by suppressing eukaryotic elongation factor 2 kinase (eEF2K) expression (Wu S. et al., 2020b), whereas LINC01619 overexpression enhances cell viability in SPCA1 cells and alleviates oxidative stress in podocytes (Bai et al., 2018; Liu Z. et al., 2020c). Thus, the downregulation of LINC01619 and upregulation of LINC00278 suggest an inflammatory and/or low survival state of PBMCs in severely affected patients. On the other hand, XIST suppresses the activation of the NF-κB signaling pathway and inflammasome (Ma et al., 2019), as well as promoting anti-inflammatory M2 macrophage polarization (Sun and Xu, 2019). Reduced levels of XIST in severe cases thus is highly likely to be correlated with CRS and severity in COVID-19 progression.

MALAT1 and NEAT1, whose upregulation in SARS-CoV-2 infected cell lines and in BALF from COVID-19 patients was discussed earlier, also stand out in the comparative analysis of severe vs. non-severe cases. In red blood cell depleted whole blood, severe cases have significantly higher levels of NEAT1 and MALAT1 in comparison to moderate cases, whereas there is no difference between moderate vs healthy control (Tang et al., 2020). Recurring observations were also made using BALF and PBMCs where MALAT1 and NEAT1 expression levels correlate with severity and an increase in pro-inflammatory cell types (Huang et al., 2022). Both lncRNAs are involved in macrophage and dendritic cell (DC) differentiation, enhancing pro-inflammatory cytokine production, and inhibiting their anti-inflammatory functions by sponging miRNA (Dai et al., 2018; Zhang M. et al., 2019b; Wu J. et al., 2020a; Wang and Guo, 2020). Hence, these results once again suggest the connection between aberrant expression of some cytokine-related lncRNAs in immune cells and severity of the disease.

Overall, lncRNA expression profiles differentiate not only between healthy and infected patients, but also between severe and non-severe COVID-19 cases (Summarised in Table 1). Severe cases display higher expression levels of lncRNAs involved in pro-inflammatory cytokine secretion, such as XIST, MALAT1 and NEAT1, reflecting a more inflammatory and less suppressive immune response that differentiate them from mild cases.

**TABLE 1:** Summary of lncRNAs undergoing differential expression in non-severe and severe COVID-19 cases. Relative levels of up and downregulation of the lncRNA are presented by + and −, respectively.

lncRNA	Non-severe	Severe	Sample type	Function of lncRNA	Reference
NEAT1	++	++++	PMBCs, NBHE and BALF	Activates M1 macrophage; directs DC differentiation; enhances pro-inflammatory cytokine secretion	(Tang et al., 2020; Vishnubalaji et al., 2020; Moazzam-Jazi et al., 2021; Huang et al., 2022)
MALAT1	++	++++	PMBCs, NBHE and BALF		
XIST	++++	+	PBMCs	Supresses NF-κB activation and NLRP3 inflammasome; promotes M2 macrophage differentiation	Cheng et al., 2021
LINC01619	+++	+	PBMCs	Overexpression enhances cell viability and alleviates oxidative stress	
LINC00278	−	++	PBMCs	Promotes apoptosis	
**lncRNA**	**Infected**		**Sample type**	**Function of lncRNA**	**Reference**
CEBPA-DT	+++		PBMCs	Supresses IFN-γ expression	Moazzam-Jazi et al., 2021
GJA9-MYCBP	+++		Cell-free plasms	Increases IL-6 and IL-6R expression	Wang et al., 2022
H19	++++		BALF	Increase IL-17 and IL23 expression	Moazzam-Jazi et al., 2021
SNHG6	+		BALF	Activate TGF-β/Smad pathway by binding to UPF1	
EGOT	+++		Viral infected Calu3 cell line	Downregulates IFN-I responses	Mukherjee et al., 2021
WAKMAR2	+++		Viral infected Calu3 cell line	Limits inflammatory cytokines during wound recovery	

### LncRNA in severity-specific leukocyte signatures

Severe COVID-19 cases present several aberrant immune landscapes, such as abnormalities in their leukocyte counts, typically lymphopenia (decreased lymphocyte numbers) and neutrophilia (high neutrophil counts).

Lymphopenia is highly correlated with COVID-19 severity (Qin et al., 2020; Song et al., 2020; Wang et al., 2020; Cheng et al., 2021). This reduction covers a wide range of lymphocytes, including CD4^+^ T cells, CD8^+^ T cells, Natural Killer (NK) cells and B cells, all of which are critical for viral clearance. The exact causes of this phenomenon are unclear but could be due to the dysregulated serum levels of TNF, IL-6 and IL-10 (Diao et al., 2020). Morenikeji et al. identified 22 lncRNAs that regulate expression of the ten most observed cytokines in COVID-19 CRS. Among them, five out of the ten cytokines (IL-6, IL-10, CSF3, TNF and CXCL10) can be regulated by a lncRNA called non-coding RNA activated by DNA damage (NORAD) (Morenikeji et al., 2020). As a lncRNA activated by DNA damage, NORAD expression might increase due to aberrant SARS-CoV-2 replication (Morenikeji et al., 2020), and its upregulation has been shown to reduce the growth of a lymphoblastoid cell line (Wang C. et al., 2019a), suggesting a potential role of NORAD in lymphopenia. Another key feature of T cells from severe cases is the higher expression of exhaustion markers, such as programmed cell death protein-1 (PD-1) and T cell immunoglobulin and mucin domain-3 (Tim-3) (Diao et al., 2020; Song et al., 2020). They are inhibitory receptors and an indication of T cell activation, but prolonged activation causes antigen-specific T cells to enter an exhaustion state, reflected by their reduced proliferative and cytotoxic capacity. This state is often associated with chronic viral infection (Barber et al., 2006; Terawaki et al., 2011; Wherry and Kurachi, 2015). LncRNAs are involved in regulation of both PD-1 and Tim-3. For example, lnc-Tim3 binds to the intracellular domain of Tim-3 and inhibits the activation of the pathway required for IFN-γ and IL-2 secretion from CD8^+^ T cells (Ji et al., 2018). Furthermore, MALAT1 upregulates PD-1 by sponging inhibitory miR-195 (Wang Q. M. et al., 2019b), whereas Tim-3 expression can be facilitated by NEAT1 through miR-155 (Yan et al., 2019), eventually increasing apoptosis of CD8^+^ T cells. Thus, the lncRNA profile in PBMCs suggests a close correlation between lncRNA and lymphopenia in severe cases of COVID-19.

Neutrophilia, is also observed in distal parts of the lung and blood of severely affected patients (Giamarellos-Bourboulis et al., 2020; Liao et al., 2020). Similar to lymphopenia, the mechanism causing neutrophilia is complicated and poorly understood. However, the fact that severe COVID-19 cases exhibit a mixture of neutrophil precursors and dysfunctional mature neutrophils suggests a dysregulation in neutrophil development is involved (Schulte-Schrepping et al., 2020). RNA-seq of whole blood cell transcriptomes shows that myeloid RNA regulator of Bim-induced death (MORRBID, as known as CYTOR) is significantly increased in severe COVID-19 cases (Aschenbrenner et al., 2021). MORRBID is an anti-apoptotic lncRNA and prolongs neutrophil lifespans, and thus a high level of MORRBID could be strongly corelated with neutrophilia (Kotzin et al., 2016). Neutrophils counteract infection by releasing neutrophil extracellular traps (NETs), but its excessive presence and NETosis, neutrophils cell death in the process of releasing NET, can also damage the surrounding tissue (Weiss, 1989).

In summary, in addition to the CRS, severity of COVID-19 infection is also associated with lymphopenia and neutrophilia. Neither of these symptoms have defined causes but lncRNA dysregulation is likely to play a role. Alongside the presence of high levels of pro-inflammatory cytokines, elevated levels of NORAD, lnc-Tim3, MALAT1 and NEAT1 may negatively impact T cell proliferation and function, impairing the immune response in infected patients. The neutrophilia seen in severe COVID-19 cases may be partially caused by expression of MORRBID prolonging neutrophil survival.

## LncRNA contributors to CRS in COVID-19 cases

One of the major sources of tissue damage in SARS-CoV-2 infections comes from the immune system itself, instead of viral replication or infection. This hyperinflammation is caused by CRS, which arises due to a combination of several dysregulated processes such as NLRP3 inflammasome hyperactivation, IL-6 and IL-17 overexpression as well as delayed IFN-I response.

### Hyperactivation of the NLRP3 inflammasome

**FIGURE 2:**
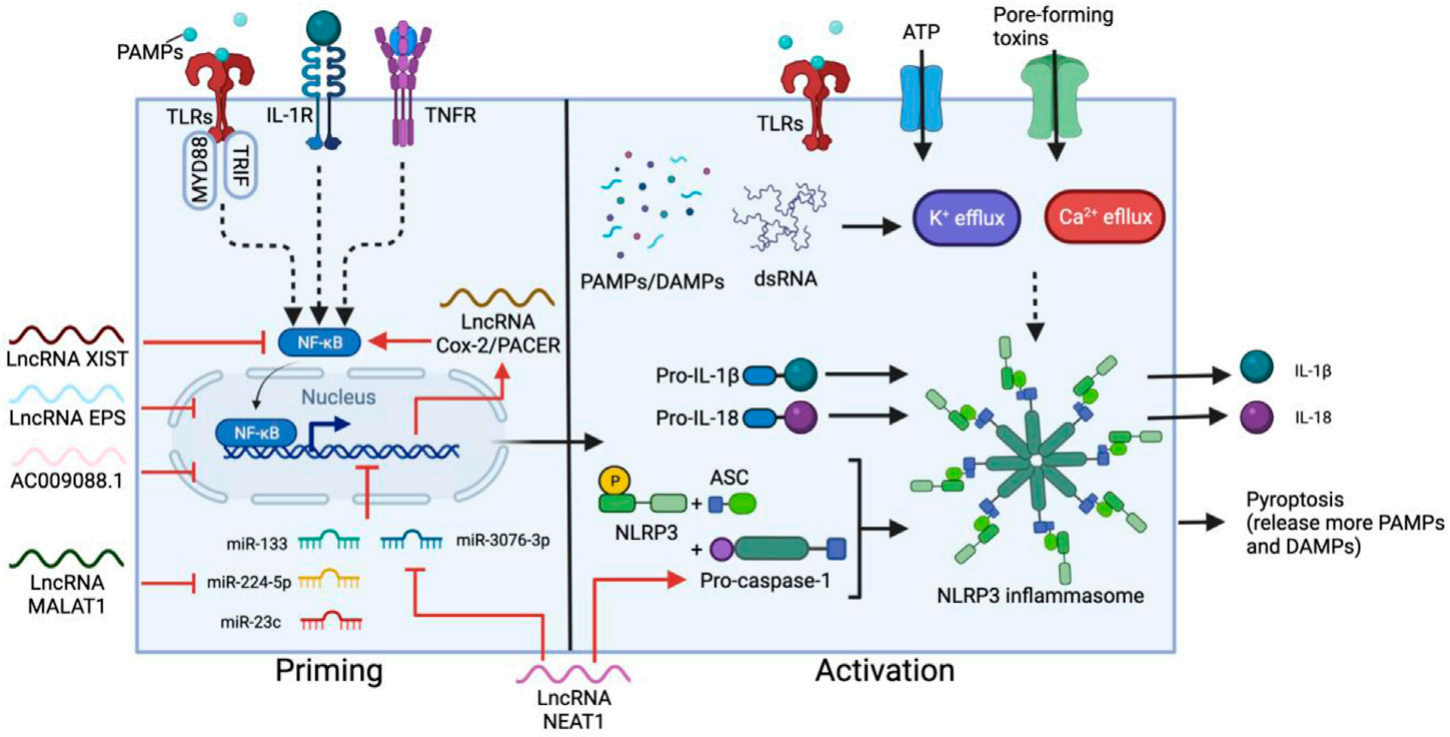
Two-step activation of NLRP3 inflammasome and the main regulatory lncRNAs. The priming step can be triggered by ligand recognition of TLRs, cytokine (such as IL-1) receptors and TNF receptors (TNFR). NF-κB is then activated by myeloid differentiation primary response 88 (MYD88) and TRIF in the TLR pathways, resulting in nuclear translocation of NF-κB and upregulation of NLRP3, pro-IL-18 and pro-IL-1β. A secondary signal, such as ATP, toxins, dsRNA, DAMPs and PAMPs stimulates the activation step, leading to the assembly of the inflammasome containing NLRP3, ASC and pro-caspace-1, which cleaves pro-IL-18 and pro-IL-1β into the mature cytokines. Most lncRNAs involved in this process regulate the priming step. XIST and PACER promotes or suppresses NF-κB activation respectively. In contrast, EPS, AC009088.1, MALAT1, and NEAT1 regulate the expression of primed genes. NEAT1 also participates in the activation step by stabilizing mature caspase-1 for inflammasome assembly.

One of the main sources of proinflammatory cytokines in CRS is the NOD-like receptor family, pyrin domain-containing 3 (NLRP3) inflammasome pathway (Lin et al., 2019; Sendler et al., 2020), which mainly functions in monocytes and is an important component of innate immune system activation. The NLRP3 inflammasome is an intracellular multimeric protein complex consisting of an NLRP3 sensor, an apoptosis associated speck-like protein containing CARD (ASC) adaptor and enzymatic subunit caspase-1. Upon auto-proteolytic activation, caspase-1 cleaves and activates the precursors of cytokines, including pro-IL-1β and pro-IL-18, triggering the downstream cytokine cascade and pyroptosis (Martinon et al., 2002) (Figure 2). NLRP3 inflammasome activation requires a priming step and activation step. Priming involves signal recognition by Toll-like receptors (TLRs) and cytokine receptors such as IL-1 and TNF receptors, followed by nuclear translocation of NF-κB to upregulate the expression of downstream genes, primarily NLRP3, pro-IL-18 and pro-IL-1β. The second step is triggered by a wide range of danger/pathogen-associated molecular patterns (DAMPs/PAMPs) (Bauernfeind et al., 2011; Franchi et al., 2014; Kelley et al., 2019) and K^+^/Ca^2+^ fluxes (Kelley et al., 2019), which induce conformational changes in NLRP3 (Meng et al., 2009), leading to inflammasome assembly, and the subsequent maturation of ILs.

Patients with severe COVID-19 can progress to ARDS, which is usually accompanied by inflammatory biomarkers, especially IL-1β, IL-6, IL-8, IL-18 and TNF (Wilson and Calfee, 2020). Many papers have addressed the association of elevated plasma IL-18 level with mortality in ARDS (Makabe et al., 2012; Dong et al., 2019; Rogers et al., 2019), emphasizing the connection between NLRP3 inflammasome and COVID-19 progression. Indeed, accumulating evidence has shown that the Spike (S) protein from SARS-CoV-2 can trigger IL-1β secretion from the NLRP3 inflammasome in macrophages (Theobald et al., 2021) and increase NF-κB nuclear translocation, NLRP3 expression levels and caspase-1 activity in PBMCs (Olajide et al., 2021). Meanwhile, the ssRNA from SARS-CoV-2 triggers a greater inflammatory response than SARS-CoV-1 and HIV-1 (Campbell et al., 2021), as cell death induced by these cytokines also releases more PAMPs/DAMPs, thus leading to both local and systematic NLRP3 hyperactivation as well as a positive feedback loop of cytokine signaling, which results in eventually lethal damage to the whole body (Channappanavar et al., 2016; Fu et al., 2020).

LncRNAs play a key role in regulating NLRP3 activation, especially at the priming step. In M1 macrophages, the expression of p50-associated cyclooxygenase-2 extragenic RNA (PACER, also known as lnc-Cox-2) is induced by the TLR receptor pathway (Ye et al., 2018). PACER removes the repressive subunit of the NF-κB complex and promotes NF-κB nuclear translocation, thus modulating the downstream expression of NLRP3, ASC and cytokine precursors (Krawczyk and Emerson, 2014; Xue et al., 2019). Knockdown of PACER reduces caspase-1 activation and IL-1β secretion from macrophages, confirming the correlation between this lncRNA and NLRP3 activation (Xue et al., 2019). Another example is lncRNA MALAT1. By sponging inhibitory miRNA, such as miR-133, miR-23c and miR-224-5p, MALAT1 overexpression enhances NLRP3 inflammasome activity and promotes macrophage pyroptosis (Li et al., 2017; Yu et al., 2018; Du et al., 2020), while silencing MALAT1 inhibits the secretion of pro-inflammatory cytokines such as TNF, IL-6, and IL-1β (Dai et al., 2018). NEAT1 also mediates the activation of the NLRP3 inflammasome in macrophages. NEAT1 binds and stabilizes mature caspase-1 to promote inflammasome assembly (Zhang P. et al., 2019c). Reducing the level of NEAT1 limits NLRP3 expression, as well as TNF, IL-1β and IL-6 production from M1 macrophages (Zhang P. et al., 2019c; Li Y. et al., 2020b; Wang and Guo, 2020). NEAT1 expression can also influence the NLRP3 inflammasome by directing DC differentiation. In DCs, NEAT1 competes with miR-3076-3p, which downregulates the expression of both NEAT1 and NLRP3 inflammasome-related genes such as NLRP3, caspase-1, and ASC (Zhang M. et al., 2019b). Knockdown of NEAT1 induces a tolerogenic phenotype in DCs which produces anti-inflammatory cytokines and suppresses the expression of NLRP3 and IL-1β (Zhang M. et al., 2019b).

There are also negative lncRNA regulators that inhibit NLRP3 inflammasome activation and are commonly expressed in unstimulated or resting macrophages/DCs. The lncRNA erythroid prosurvival (EPS) and lncRNA AC009088.1 inhibit the expression of ASC, encoded by the *Pycard* gene. EPS binds to regulatory regions of its target genes to help maintain an epigenetic repressive state and reduce RNA pol II accessibility in resting macrophages (Atianand et al., 2016). Conversely, AC009088.1 is a *cis*-acting reverse transcript of *Pycard* (Moazzam-Jazi et al., 2021) and might act in a similar way as another *Pycard* antisense lncRNA, PYCARD-AS1, which binds *Pycard* mRNA and prevents ribosome assembly and protein translation from occurring (Miao et al., 2019). In addition, a stimulatory effect of lncRNA XIST knockdown on the expression of NLRP3, TNF, IL-1β, IL-8 and IL-6 is observed bovine cell models (Ma et al., 2019). The absence of XIST enhances phosphorylation levels of the p65 subunit of NF-κB, thus promoting NF-κB activation and downstream gene expression. The inhibitory effect of XIST may also related to its function of silencing one of the X-chromosomes in females. Strickland et al. linked improper X-chromosome inactivation to elevated expression of immune response genes in autoimmune disorders, as many genes encoded on X-chromosomes are related to CD4^+^ T cell and macrophage activation (Strickland et al., 2012).

Interestingly, many of the NLRP3-regulating lncRNAs undergo changes in expression levels during SARS-CoV-2 infection. The positive regulators such as MALAT1 and NEAT1 are increased in expression, whereas the levels of inhibitory lncRNAs such as XIST and AC009088.1 are downregulated in PBMCs (Moazzam-Jazi et al., 2021). Furthermore, the effects of MALAT1, NEAT1 and XIST are correlated with COVID-19 severity. Such consistency between lncRNA mechanisms and clinical observations suggests the potential of lncRNAs as diagnostic or possibly predictive biomarkers for SARS-CoV-2 infection.

### Serum IL-6 elevation

Although IL-6 is not the sole contributor to the CRS, studies have found that levels of IL-6 are strongly correlated with patients’ clinical severity, including severe lung injury and high mortality rates (Coomes and Haghbayan, 2020). Meta-analysis found that the mean IL-6 concentration in severe cases is ∼2.9-fold higher than non-severe cases (Coomes and Haghbayan, 2020). Furthermore, severe COVID-19 symptoms can be alleviated by drugs such as Tocilizumab that blocks IL-6 binding to its receptor on cell surfaces (Liu B. et al., 2020a; Angriman et al., 2021).

**FIGURE 3:**
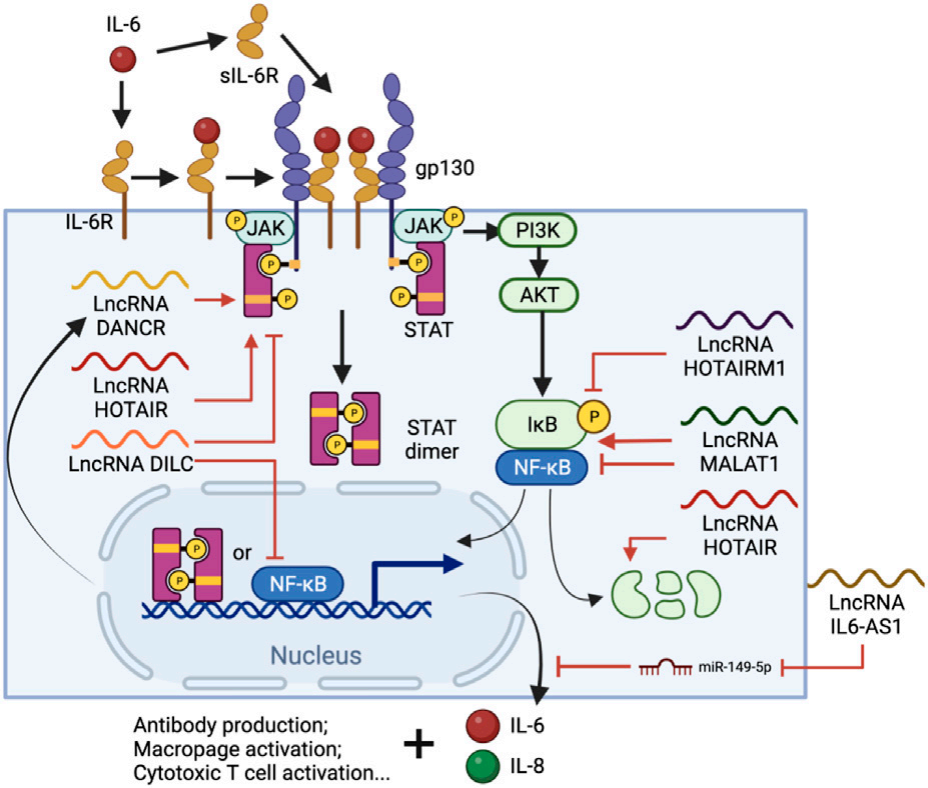
IL-6 signal cascade and regulatory lncRNA involved in the process. IL-6-bound IL-6R or sIL-6R forms complexes with membrane protein glycoprotein 130 (gp130), which triggers the JAK/STAT pathway and eventually leads to STAT3 phosphorylation and nuclear translocation. NF-κB is also activated via the (PI3K)-AKT pathway and, together with STAT3, promotes immune cell differentiation and IL-6, IL-8 expression. Throughout the process, lncRNAs such as DANCR, HOTAIR, HOTAIRM1, DILC and MALAT1 are involved in either or both pathways by regulating STAT3 phosphorylation, NF-κB interacting with chromatin or the degradation of IκB. Meanwhile, IL-AS1 can regulate IL-6 expression by sponging miR-149-5p, thus protecting IL-6 mRNA.

IL-6 is a pivotal cytokine secreted from several types of immune cells, especially T cells and monocytes. IL-6 is one of the most important cytokines during viral infection, along with IL-1 and TNF-ɑ (Dienz and Rincon, 2009). It stimulates antibody production in B cells and also the differentiation of macrophages and cytotoxic T cells (Tanaka T. et al., 2014b). However, IL-6 also inhibits the development of regulatory T cells which modulate effector T cell function (Kimura and Kishimoto, 2010). Upon ligand binding, IL-6 receptors (IL-6R) initiates a signal cascade through the Janus kinase/signal transducer and activator of transcription (JAK/STAT) pathway (Wang et al., 2013), which also triggers phosphatidylinositol 3-kinase (PI3K)-AKT and Ras-dependent pathways to activate NF-κB (Nakafuku et al., 1992; Yamada et al., 2012), facilitating the expression of pro-inflammatory cytokines, proliferation-related genes, and more IL-6 (Tanaka et al., 2016) (Figure 3). Furthermore, IL-6 activates the extracellular soluble form of IL-6R (sIL-6R), which can trigger the IL-6 cascade in surrounding cells, causing systemic cytokine responses, autoimmune responses and chronic inflammation (Jones et al., 2001). Treatment with SARS-CoV-2 S protein increases IL-6 up to 7-fold in human PBMCs (Dosch et al., 2009), and 50-fold in murine macrophages through NF-κB-mediated transcriptional activation (Wang et al., 2007). However, S protein not only stimulates the release of IL-6, but also sIL-6R, enhancing the hyper-inflammatory response and weakening the effect of Tocilizumab by bypassing the need for IL-6R binding (Patra et al., 2020; Chen et al., 2021).

IL-6 is regulated by multiple factors including lncRNAs (Figure 3). Some lncRNAs act on both or either NF-κB and/or JAK/STAT pathways to regulate IL-6 production. A lncRNA called downregulated in liver cancer stem cells (DILC), for example, has a suppressive role in both pathways. Knockdown of DILC increases STAT3 phosphorylation and tumor propagation, possibly through blocking the autocrine activity of IL-6. This lncRNA also competes with NF-κB to bind the IL-6 promoter and thus inhibits IL-6 transcription at high expression levels (Wang et al., 2016). In contrast, the lncRNA called HOX antisense intergenic RNA (HOTAIR) upregulates IL-6 via both pathways. HOTAIR facilitates the degradation of IκBα, an inhibitor of NF-κB nuclear translocation, and thus activates NF-κB and its downstream gene transcription as well as IL-6 release from macrophages (Obaid et al., 2018). HOTAIR also interacts with PRC2 and enhancer of zeste homolog 2 (EZH2), the lysine methyltransferase subunit of PRC2, which binds and methylates STAT3, leading to enhanced tyrosine phosphorylation and activity of STAT3 (Gupta et al., 2010; Kim et al., 2013). IL-6 antisense RNA 1 (IL6-AS1) is another IL-6 upregulator and promotes the expression of IL-6 by sponging miR-149-5p to stabilize IL-6 mRNA and recruiting epigenetic modifiers to increase active H3K4me3 and H3K27ac at the IL-6 promoter (Yi et al., 2021).

Interestingly, many lncRNAs which are differentially expressed in COVID-19 patients’ BALF were heavily involved in lncRNA-mediated regulation of IL-6. HOTAIR myeloid 1 (HOTAIRM1), for example, undergoes a dramatic reduction of expression in COVID-19 patients (Moazzam-Jazi et al., 2021). HOTAIRM1 reduces phosphorylation of p65 and IκBα, inactivating the NF-κB pathway (Ren et al., 2021), thus its reduction is consistent with the observation of high IL-6 level in the patients. Positive regulatory lncRNAs such as differentiation antagonizing nonprotein coding RNA (DANCR), on the other hand, are expressed at a higher-level during SARS-CoV-19 infection (Moazzam-Jazi et al., 2021). DANCR transcript levels increase upon IL-6 stimulation in a STAT3-dependent manner, but itself also promotes the interaction between STAT3 and JAK1, thus amplifying IL-6 production and signaling (Zhang X. et al., 2019d), forming a positive feedback loop that keeps enhancing inflammation in the local area. However, the effects of some lncRNAs can be more complicated. Knockdown of MALAT1 upregulates NF-κB and IL-6 expression in renal ischemia-reperfusion injury (Tian et al., 2018), but also inhibits IL-1β, IL-6 and TNF-ɑ production in Lipopolysaccharide (LPS)-induced acute lung injury (Dai et al., 2018), suggesting a duel function of this lncRNA in inflammatory regulation.

### High IL-17 levels and ORF8 hijacking

IL-17 (IL-17A) is another cytokine that dramatically increases in ARDS caused by SARS-CoV (Muir et al., 2016), MERS-CoV (Mahallawi et al., 2018), and SARS-CoV-2 (Hasan et al., 2021). IL-17 is a pivotal cytokine mainly produced by T helper 17 (T_H_17) cells (Harrington et al., 2005; Park et al., 2005), and is commonly known for its role in neutrophils differentiation and recruitment, thus high IL-17 level might contribute to neutrophilia in severe COVID-19 patients.

**FIGURE 4:**
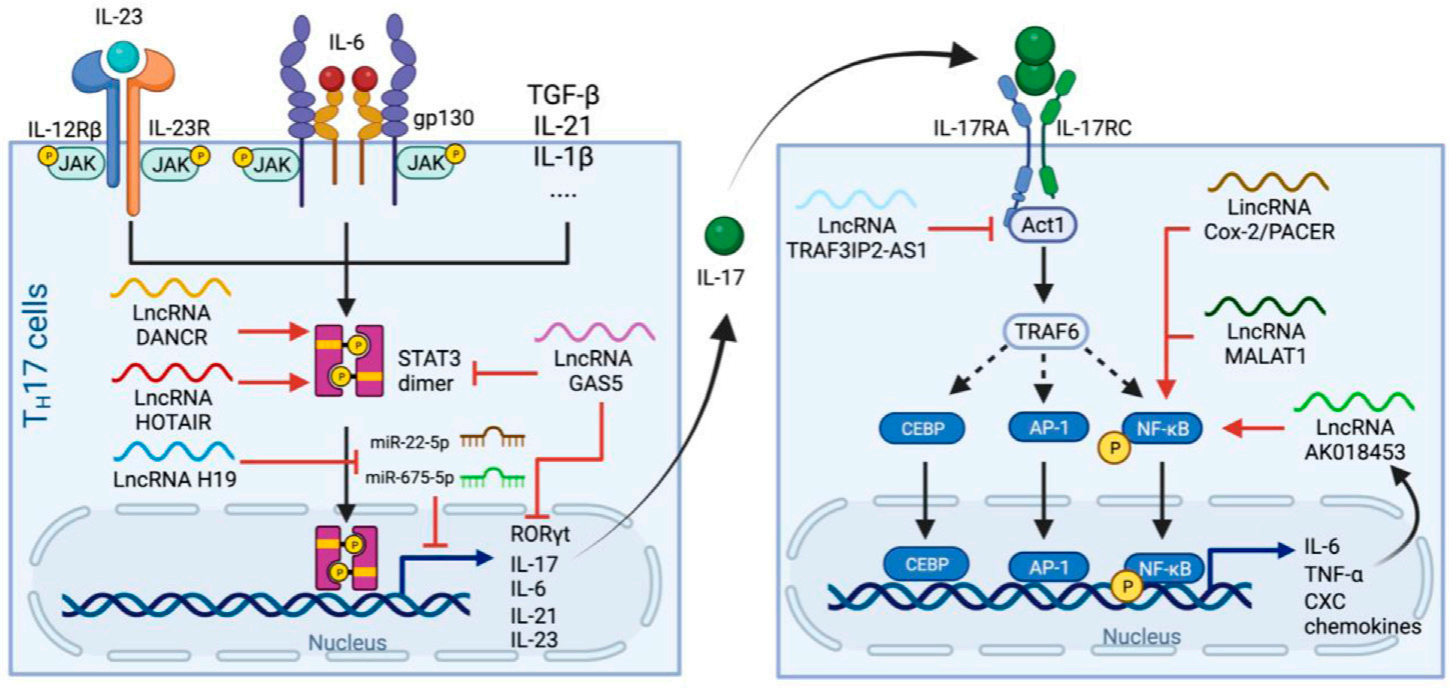
Production and signaling pathways of IL-17 and regulatory lncRNAs in the process. *Left panel.* T_H_17 cells differentiate upon receiving IL-23, IL-6 and other cytokine signaling. Activated IL-6 and IL-23 receptors leads to phosphorylation and dimerization of STAT3 which subsequently initiates the expression of genes related to T_H_17 programming. LncRNAs such as DANCR and HOTAIR stimulate STAT3 dimerization in a similar manner to that of in the IL-6 pathway, whereas H19 sponges miR-22-5p and miR-675-5p to stimulate IL-17 production. GAS5 inhibits T_H_17 differentiation by promoting STAT3 degradation and suppresses RORγt and IL-17 expression. *Right panel.* IL-17 from T_H_17 cells dimerizes and binds to IL-17RA/C, which activates Act1 and then TRAF6 to facilitate nuclear translocation of CBEP, AP-1 and NF-κB and thus the release of IL-6, TNF-⍺ and CXCs. This pathway also induces the expression of the lncRNA AK018453, which, together with PACER and MALAT1, enhances NF-kB-mediated IL-17-dependent responses. Whereas TRAF3IP2-AS1 downregulated the expression of Act1 and suppress the effect of IL-17.

T_H_17 cell differentiation is defined by the expression of retinoic acid receptor-related orphan nuclear receptor γt (RORγt), a transcription factor essential for T_H_17 programming and IL-17 production (Ivanov et al., 2006). The secreted IL17 dimerizes and signals through IL-17 receptor A and C subunits (IL-17RA/C). IL-17RA/C initiates the downstream cascade via adaptor molecule Act1 which recruits TNF receptor associated factor 6 (TRAF6) to activated NF-κB pathway (Wu et al., 2012). Transcription factors activator protein 1 (AP1) and CEBP also translocate into nucleus and, together with NF-κB, induce the expression of many pro-inflammatory cytokines such as IL-6 and TNF-⍺, as well as neutrophil-specific CXC chemokines (Zenobia and Hajishengallis, 2015) (Figure 4).

Similar to the cases of NLRP3 and IL-6, many lncRNAs involved in IL-17 pathways also undergo differential expression during SARS-CoV-2 infection. For example, H19 is one of the most overly expressed lncRNA in patient’s BALF (Moazzam-Jazi et al., 2021), and its overexpression increases the production of IL-17 (and also IL-23) by mediating miR-22-5p and miR-675-5p (Zhang et al., 2020). In contrast, an anti-inflammatory lncRNA, growth arrest-specific 5 (GAS5), is expressed at a lower level in severe cases comparing to non-severe cases (Cheng et al., 2021). GAS5 overexpression reduces the level of RORγt, possibly through promoting ubiquitination and subsequent degradation of STAT3 (Li J. et al., 2020a), suggesting that the high T_H_17/IL-17 level and neutrophilia in severe cases might be related to their low GAS5 level.

Noteworthy, the treatment of COVID-19 using IL-17 inhibitor such as Secukinumab (human monoclonal antibody to IL-17) did not improve patients’ hospitalization time and intensive care unit demand (Resende et al., 2022), possibly because IL-17R can also be hijacked by SARS-CoV-2 ORF8 protein, which interacts with IL-17RA and activates the pathway in a stronger and broader manner than IL-17 (Lin et al., 2021; Wu et al., 2022). Therefore, it might be more efficient to restrict the outcome the IL-17 overexpression by interfering in the downstream pathways of IL-17R rather than the release of IL-17. One potential target is AK018453, an IL-17-induced lncRNA that carries out the effect of IL-17 by promoting gene expression through TRAF1/Smad pathway. AK018453 knockdown was also reported to reduce pro-inflammatory cytokine production in the IL-17-treated astrocytes (Zhang et al., 2022). Another example, TRAF3IP2-AS1, is the antisense lncRNA of the gene encoding for Act1 (*TRAF3IP2*) and negatively regulates IL-17 signal by downregulating the transcription factor essential for Act1 expression (He et al., 2021).

Overall, the involvement of lncRNAs in both IL-17 production and downstream pathways suggest that they can both be used as biomarkers and/or therapeutic targets in order to bypass SARS-Cov-2 hijacking.

### Impaired type I IFN (IFN-I) response

Hyperactivation of the NLRP3 inflammasome and elevated IL-6 are commonly seen in CRS induced by a number of viruses, such as avian influenza, SARS-CoV-1, MERS-CoV and Ebola virus (Teijaro, 2017). However, the cytokine response triggered by SARS-CoV-2 is different to other CRS-inducing viruses, as it does not trigger as much secretion of IL-2, IL-10, IL-4, or IL-5 and the IFN-I response is much delayed (Hadjadj et al., 2020; Olbei et al., 2021).

**FIGURE 5:**
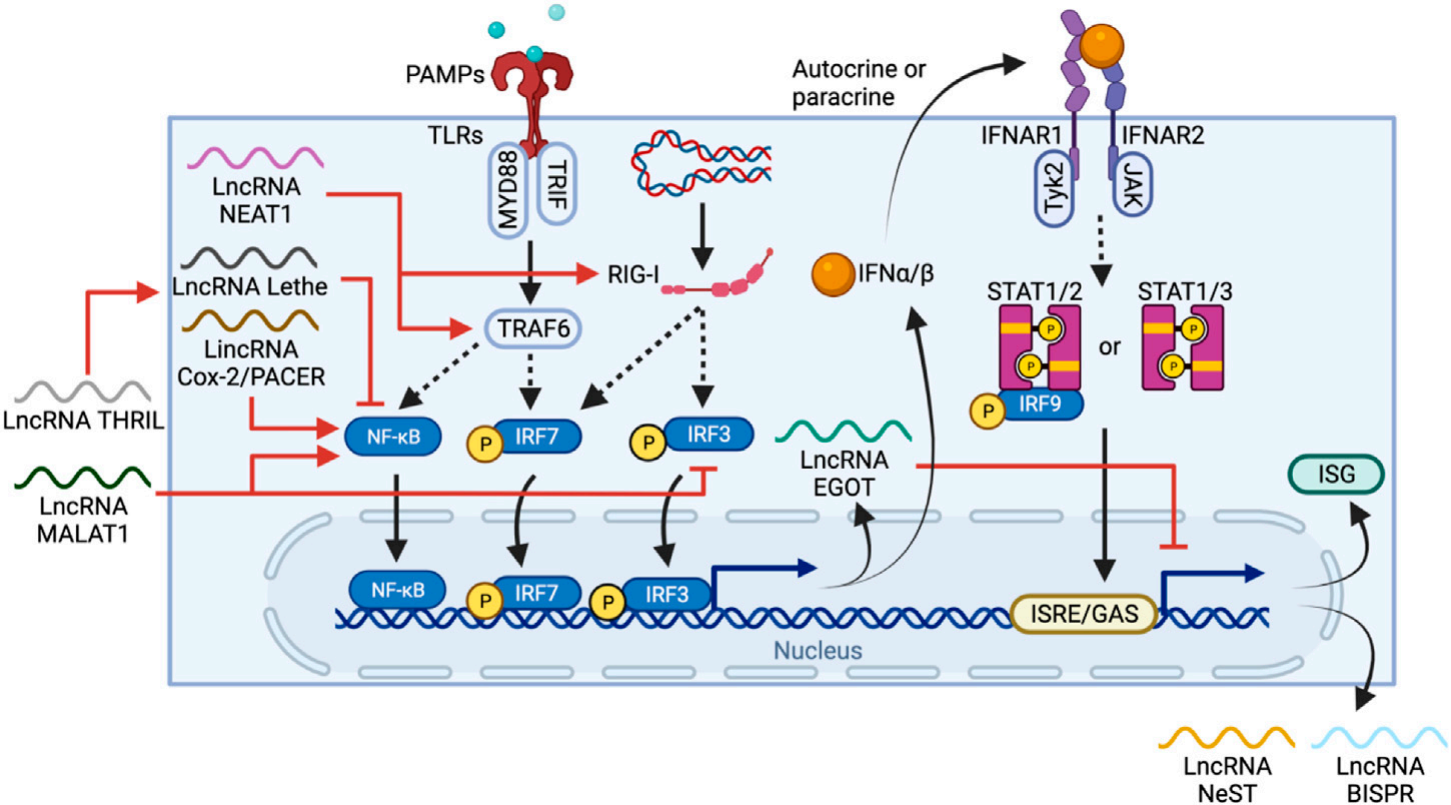
LncRNA regulation and feedback loops in IFN-I production and ISG expression. PAMPs or foreign nucleic acid are sensed by TLRs and RIG-I, respectively. The TLR pathway activates IRF6 and NF-κB through TRAF6, whereas RIG-I activates IRF3/7 directly, leading to their nuclear translocation and IFN-I expression. IFN-I can then stimulate IFNAR in either autocrine or paracrine manners to trigger a STAT1/2/3-mediated pathway, promoting ISG expression. LncRNAs regulate one or more elements in the IFN-I production cascade, including activation of TRAF6 and RIG-I by NEAT1, activation of NF-κB by MALAT1 and PACER, or inhibition of NF-κB by THRIL and Lethe. EGOT forms a negative feedback loop, itself is induced by TLR and RIG-1 pathways but also regulates IFN-1 induced responses by inhibiting the expression of ISG and other anti-viral lncRNAs such as NeST and BISPR.

IFNs, especially type I, are crucial cytokines in the immune defense against viral infection. IFN-I consists of several IFN-ɑ subtypes and IFN-β, which are all secreted by a number of cells, including lymphocytes, macrophages and endothelial cells. PAMPs, LPS or foreign nucleic acid sensed by TLRs and cytosolic receptors, such as retinoic acid-inducible gene I (RIG-I), trigger a signal cascade that activate TRAF6 and eventually phosphorylates IFN-regulatory factor 3/7 (IRF3/7) or NF-κB to induce expression of IFNs (McNab et al., 2015). IFNɑ/β can also act in an autocrine or paracrine manner to initiate expression of IFN-stimulated genes (ISGs) by binding to IFN-I receptors (IFNAR) on a wide range of cell types, including macrophages, DCs, and NK cells (de Weerd and Nguyen, 2012). The IFNAR is a heterodimer of the proteins IFNAR1 and IFNAR2. IFN-I binding to the IFNAR causes activation of tyrosine kinases 2 (TYK2) and JAK1, which subsequently phosphorylate STAT1/2/3. Activated STAT1 and two dimerize and interact with IRF9 to form ISG factor 3 (ISGF3) whereas phosphorylated STAT1 or STAT3 forms homodimers and induces ISG expression by binding to corresponding DNA IFN-stimulated response elements (ISREs) or γ-activated sequences (GAS) (Figure 5) (Ivashkiv and Donlin, 2014).

Throughout the process, lncRNAs regulate both IFN-I production and ISG expression. PACER, as described above, is induced via the TLR pathway, and promotes NF-κB nuclear translocation (Krawczyk and Emerson, 2014). In contrast, MALAT1, on top of activating NF-κB, also binds and prevents the activation of transactive response DNA-binding protein (TDP43) which inhibits IRF3 degradation, thus negatively regulating IFN-I (Liu W. et al., 2020b). The lncRNA called TNF-α and hnRNPL-related immunoregulatory lincRNA (THRIL) can indirectly downregulate IFN-I expression by promoting TNF expression (Li et al., 2014), which in turn induces expression of another lncRNA Lethe, an inhibitor of NF-κB DNA-binding activity (Rapicavoli et al., 2013).

Feedback loops are also commonly seen in lncRNA-mediated regulation of IFN-I. For example, NEAT1 is upregulated through the RIG-1-IRF7 pathway upon viral infection and forms a positive feedback loop that enhances IFN-β production by increasing RIG-I expression (Ma et al., 2017). In contrast, EGOT is expressed via RIG-I and TLR/NB-κB pathways, but in turn, suppresses the antiviral responses induced by IFN-I (Carnero et al., 2016). Thus, the high level of MALAT1, NEAT1 and EGOT observed in COVID-19 patients potentially work together and are correlated to the delayed IFN-I response (Moazzam-Jazi et al., 2021). However, due to the lack of lncRNA profiling of COVID-19 patients at early vs late stages of infection, evaluating the contribution of lncRNA is challenging.

Common viral infections can quickly induce IFN-I production, but many coronaviruses have evolved to inhibit IFN-I response, including SARS-CoV-1, MERS-CoV and SARS-CoV-2 (Totura and Baric, 2012; Lau et al., 2013; Channappanavar et al., 2016). SARS-CoV-2 can interfere with the IFN-I expression pathway using multiple structural and non-structural proteins. ORF3b of SARS-CoV-2, for instance, can inhibit IFN-I production by impairing nuclear translocation of IRF3, and is more potent than its SARS-CoV-1 ortholog (Konno et al., 2020). Furthermore, SARS-CoV-2 ORF6 and Nsp1 protein not only impede IRF3 translocation and IFN-I production, but also the downstream IFN-β-induced ISG expression by inhibiting STAT1/2 phosphorylation and nuclear translocation (Lei et al., 2020; Miorin et al., 2020; Thoms et al., 2020). This disrupted IFN-I timeframe causes several negative impacts on the downstream immune system and is often associated with disease severity (Blanco-Melo et al., 2020; Hadjadj et al., 2020). Firstly and most fundamentally, attenuation of IFN-I production suppresses the host’s ability to restrict viral replication at early stages of infection (Channappanavar et al., 2016). IFN-I induces many anti-viral lncRNAs such as BST2 IFN-stimulated positive regulator (BISPR), which helps prevent the release of viral particles from cells (Barriocanal et al., 2014), as well as Nettoie *Salmonella* pas Theiler’s (NeST), which upregulates IFN-γ expression from NK cells (Gomez et al., 2013). If this limitation on viral replication is insufficient, the subsequent cell death and local inflammation caused induce additional cytokines and recruit more immune cells into the lung (Rodriguez and Brodin, 2020). Moreover, IFN-I epigenetically reprograms TNF-induced tolerance in monocytes, restoring their responses to certain TLR signals and enhancing their inflammatory responses (Park et al., 2017). This is possibly due to the feedback loops between ISG expression and IFN-induced lncRNA, as some lncRNAs such as EGOT are suppressive and prevent hyperactivation of immune responses. Thus, high levels of other cytokines combined with a delayed IFN-I signal cause accumulated activation of monocytes as well as their cytokine production. And lastly, because IFN-I is still released at later stages of infection, it could interfere with lncRNA expression control. Indeed, its concentration was found to correlate with COVID-19 severity (Contoli et al., 2021). For example, MORRBID is expressed upon IFN-I stimulation (Kotzin et al., 2019), but its overexpression could lead to neutrophilia (Kotzin et al., 2016), which is more commonly observed in severe patients.

Therefore, SARS-CoV-2 proteins disrupt the timing of IFN-I response by inhibiting its release at beginning phase of infection. This effect restricts patients’ early virus clearance ability and when IFN-I production peaks, it coincides with the recruitment and activation of monocytes. An increase in SARS-CoV-2 viral load enhances inflammatory cytokine release and suppresses monocytes leading to atypical CRS and increasing risk of severity.

## LncRNA as biomarkers and/or therapeutic targets for COVID-19

LncRNAs are participants in crucial pathways of regulating host immune responses and cytokine secretion, and some of them display significant changes during disease stage transition and progression, and thus many papers have suggested using lncRNA profiles as biomarkers to predict the infection progression or as therapeutic targets for COVID-19 (Cheng et al., 2021; Yang et al., 2021).

The lncRNA profiles among healthy, non-severely affected and severely affected patients have distinct characteristics. NEAT1 and MALAT1 are two of the most significantly changed due to their involvement in multiple inflammatory pathways. The fact that NEAT1 and MALAT1 upregulation is more pronounced in severe cases than in mild and moderate cases also highlights their potential as biomarkers. In addition, these two lncRNAs are highly present not only in patient’s lung samples and PBMCs but also saliva and nasopharyngeal swab samples, making them easily accessible for daily testing and monitoring (Rodrigues et al., 2021). However, both of these lncRNAs have been proposed as biomarkers for various types of cancer (Li et al., 2018; Thankachan et al., 2021), which calls their specificity as biomarkers for COVID-19 into question. Therefore, it is necessary to combine them with other clinical and biochemical signatures, for instance by adding profiles of multiple lncRNAs as one of the values into the COVID-19 scoring system and risk stratification.

The changes in lncRNA and immunopathology of COVID-19 are likely to be correlated both in time and space, thus using lncRNAs as predictive markers of disease severity may have limited application. Instead, lncRNA profiles are a good reflection of physiological and pathological conditions of the patients and can act as a guide for the corresponding therapy. Such a strategy is more commonly seen in cancer treatment. For example, the mRNA-lncRNA signature in triple negative breast cancer is currently undergoing a clinical trial as a biomarker of chemotherapy efficacy (www.clinicaltrials.gov; identifier NCT02641847). For COVID-19, an abnormal expression pattern of lncRNAs related to T cell differentiation/activation could be a potential indicator for T-lymphopenia and requirement for modulating effector vs regulatory T cell balance, which could lead to better outcomes of in treatment and recovery (Zheng et al., 2020). Moreover, some lncRNAs and miRNAs are also found to be associated with SARS-CoV-2 infection-induced male infertility/reproductive disorder, which is a less immediate outcome of the infection and these ncRNAs could serve as early diagnostic biomarkers, thus encouraging early treatment (Sabetian et al., 2021).

Therapeutically, lncRNAs could be used to suppress CRS in COVID-19 patients. LncRNAs are heavily involved in NLRP3 inflammasome activation and dysregulation of IL-6 signaling pathways that are both correlated with CRS. This makes lncRNAs ideal targets for manipulating the immune response during COVID-19. For example, by adjusting the timing of the lncRNA expression with medications, clinicians might be able to re-initiate IFN-I production at early infection and reduce prolonged proinflammatory responses. Some anti-inflammatory drugs use similar approaches, such as emodin, which upregulates the lncRNA TUG1 to attenuate inflammation induced by LPS (Liang and Ren, 2018). However, there are still other factors that need to be considered. First is specificity: one lncRNA targets more than one gene/transcript/biological pathway in the immunity network, such as MALAT1 being a dual regulator of IL-6 in different tissues and an upregulator of both protective IFN-I and proinflammatory NLRP3 inflammasome. The lncRNA and tissue of choice need to be carefully considered to ensure a successful therapy. Secondly, three strategies have been proposed for lncRNA treatment in cancer (Wang W. T. et al., 2019c), but all involve a RNAi-like approach; that is, using single-stranded antisense oligonucleotides with specific complementarity to promote target RNA degradation by RNase H, with differences mainly existing in the nucleotide type (RNA/DNA/locked nucleotides) and the oligo lengths. So far, these strategies have been applied to silence or suppress lncRNAs such as MALAT1 in mouse models of myotonic dystrophy type 1 (Wheeler et al., 2012). Lastly, the method of delivery must be considered. Several RNA carrier systems have been extensively investigated, including nanoparticles and ncRNA modification, to varying degrees of success. Nanoparticles can be either lipid-based, inorganic or polymeric, and lipid nanoparticles have been already used for mRNA delivery in many clinical trials/therapies, including for COVID-19 vaccines (Hou et al., 2021). Using modified nucleotides is another way to improve lncRNA durability and targeting precision. For example, chemically conjugated RNA with (2–3)N-acetylgalactosamine (GalNAc) can greatly increase the delivery efficiency (Nair et al., 2014). Adding 2′-O-methyl and 2′-deoxy-2′-fluoro ribose modifications can further stabilise siRNA (Foster et al., 2018). Currently, the number of clinical trials involving lncRNAs is still significantly lower than other therapies. Given the involvement of lncRNAs in the immune response to and pathogenesis of SARS-CoV-2 infection, evaluation of their therapeutic and diagnostic potential is warranted. However, further studies are still required for practical lncRNA application in COVID-19 and other diseases.

In conclusion, lncRNAs have been gaining popularity as a new research, clinical, and therapeutic target. In this review, we discussed the lncRNA expression profiles in both non-severe and severe COVID-19 cases, as well as the involvement of lncRNAs in lymphopenia, neutrophilia, NLRP3 activation, IL-6 dysregulation, and IFN-I response delay. LncRNAs expression pattern shows high consistency with patient’s infection severity and other immune landscapes, suggesting the value of comparing lncRNA profiles from patients with different severities or even at different infection stages. However, the number of this type of extensive studies is still low even in the third year into the pandemic, leaving a gap remaining to be filled. As the understanding of lncRNAs in COVID-19 infection grows, so do their potential as biomarkers for both diagnosing and evaluating a patient’s CRS state, as well as for being the therapeutic target in RNA-based vaccines and treatments.
